# Autonomous Driving of Trackless Transport Vehicles: A Case Study in Underground Mines

**DOI:** 10.3390/s25103189

**Published:** 2025-05-19

**Authors:** Yunjie Sun, Linxin Zhang, Junhong Liu, Yonghe Xu, Xiaoquan Li

**Affiliations:** 1 School of Resources Environment and Materials, Guangxi University, 100 University East Road, Nanning 530004, China; 2115302008@st.gxu.edu.cn (Y.S.); 2215302008@st.gxu.edu.cn (L.Z.); 2315394011@st.gxu.edu.cn (J.L.); 2315302008@st.gxu.edu.cn (Y.X.); 2 State Key Laboratory of Featured Metal Materials and Life-Cycle Safety for Composite Structures, Guangxi University, 100 University East Road, Nanning 530004, China; 3 Guangxi Colleges and Universities Key Laboratory of Minerals Engineering, Guangxi University, 100 University East Road, Nanning 530004, China

**Keywords:** trackless transport, light band, autonomous driving, underground mines, image recognition, metal mine, autonomous operation

## Abstract

**Highlights:**

**What are the main findings?**

- We propose an autonomous driving method for underground rubber-tired vehicles based on light band guidance.
- The feasibility of the method was validated through model experiments.

**What is the implication of the main finding?**

- This method is characterized by simplicity, practicality, strong stability, and low cost.
- The scaled-down model experiments have strong simulation capabilities, and the experiments validated the feasibility of the method, laying the foundation for future large-scale practical applications.

**Abstract:**

The introduction of autonomous vehicles in underground mine trackless transportation systems can significantly reduce safety risks for personnel in production operations and improve transportation efficiency. Current autonomous mining vehicle technology is characterized by complex algorithms and high deployment costs, which limit its widespread application in underground mines. This paper proposes a light-band-guided autonomous driving method for trackless mining vehicles, where a continuous, digitally controllable light band is installed at the tunnel ceiling to provide uninterrupted vehicle guidance. The light band is controlled by an independent hardware system and uses different colors to indicate vehicle movement status, enabling vehicles to navigate simply by following the designated light trajectory. We designed the necessary hardware and software systems and built a physical model for validation. The system enabled multiple vehicles to be guided simultaneously within the same area to perform diverse transportation tasks according to operational requirements. The model vehicles maintained a safe distance from tunnel walls. In GPS-denied environments, positioning was achieved using dead reckoning and periodic location updates at designated points based on the known light-band trajectory. The proposed method demonstrates high potential for practical applications.

## 1. Introduction

The working environment in underground mines is complex and harsh [1], characterized by low production efficiency and frequent accidents [2]. Achieving unmanned and minimally manned operations is the optimal solution to address harsh environments and ensure mining safety, making unmanned mining an inevitable trend in the industry’s future development [3]. Trackless transportation is one of the primary transport methods in underground metal mines and constitutes a vital component of mining operations. Therefore, the intelligent design of trackless mining trucks and the realization of autonomous driving are of great significance. The existing research has proposed various methods and tested them in different application scenarios.

Open-pit mines have successfully deployed unmanned mining trucks [4], achieving promising results. Based on the research achievements in ground-based autonomous driving technology and the widespread coverage of 5G networks, mining trucks employ GPS, artificial intelligence, and big data to enable accurate positioning and the detection of obstacles and lanes. The vehicles are equipped with multiple sensors to collect vast amounts of environmental information, generating gigabytes of data within seconds [5], which only 5G and higher networks can accommodate to meet such high communication standards. Due to the underground mining environment, the transmission range of 5G signals is reduced, necessitating an increased number of base stations [6,7], which exponentially raises construction costs compared with surface deployment. Vehicle positioning is fundamental to autonomous driving, but in underground mining environments, GPS cannot be used for vehicle localization [8,9], making traditional ground-based positioning methods difficult to apply. Furthermore, the highly similar characteristics of underground mining environments further increase the difficulty of achieving precise positioning. Therefore, the unmanned driving methods used in open-pit mines cannot be directly transferred to underground mines, necessitating the development of new methods and technologies tailored to underground mining environments.

Artificial beacons are often used for positioning or transmitting critical information in underground mines. Radio frequency identification (RFID) is widely used for personnel and equipment positioning [9,10], offering low cost, durability, and high security, and can function properly even when exposed to dust and muddy water. Its drawback is that when used alone in underground mine environments, its positioning accuracy is limited; thus, it is often fixed on tunnel walls to calibrate errors from other methods or placed at critical nodes, such as tunnel intersections, to provide guidance information [11,12]. Barcodes are another commonly utilized beacon type. Xu et al. [13], for example, arranged paired barcodes at fixed intervals along both sides of tunnel rock walls. Two onboard vision sensors were then used to detect each barcode pair, and a visual distance projection model was applied to compute the distance between the upper-left inner corner of each barcode feature box and the vehicle’s center. This method effectively determined the vehicle’s 3D coordinates with high accuracy. However, on bumpy roads or when barcodes are contaminated, they may fail to be recognized correctly, leading to errors. Additionally, when modifications are required, the aforementioned artificial beacons must be manually redeployed, lacking flexibility in adjustment.

At present, the navigation and localization of autonomous mobile mining equipment are mainly based on the use of LiDAR. Chi et al. [14] used two 2D LiDAR sensors to measure the distance to the tunnel rock walls and calculate the vehicle’s heading angle for real-time adjustments, while barcode beacons were employed to correct localization errors. Vasilis Androulakis et al. [15] employed four 2D LiDAR sensors for environmental mapping and four ultrasonic sensors for short-range safety detection. The RANSAC algorithm was used to extract pillar rib features from the LiDAR data, and a Stanley controller was introduced to adjust the steering angle, ensuring that the shuttle vehicle remained centered on the roadway. In simulation experiments, the minimum success rate reached 84%. In these studies, researchers used LiDAR data to determine the vehicle’s relative position within the tunnel for autonomous navigation, but errors were likely when encountering obstacles or turning points. SLAM (simultaneous localization and mapping) technology is the mainstream strategy for positioning unmanned mining equipment in underground mines, particularly suitable for enclosed environments where GPS is unavailable. In early research, 2D LiDAR was used as the primary sensor for SLAM navigation, but it was insensitive to vertical tunnel features [8,16]. Road undulations induced pose uncertainty, resulting in mapping inaccuracies. SLAM based on 3D LiDAR is currently a research hot spot and is considered to have greater application potential in underground mining environments. The 3D LiDAR is capable of providing three-dimensional environmental information, including height variations at observation points and environmental dynamics [17]. Additionally, it has a lower probability of map matching errors, although it comes at a higher cost [18]. Mine rescue robots built on 3D SLAM technology possess high mapping and positioning accuracy, enabling autonomous navigation in collapsed tunnels. The application of particle filtering in such devices significantly improves the accuracy of pose estimation [19]. Frosi Matteo et al. [20] developed a 6DoF SLAM system that incorporates an IMU module, achieving high-precision pose estimation and mapping without requiring loop closure. Solid-state LiDAR is used for localization and mapping in tunnel environments, offering high accuracy and a significant cost advantage over mechanical LiDAR. Wang et al. [8] used a hardware system consisting of a mobile mining vehicle, solid-state LiDAR, and an IMU, achieving precise mapping of underground tunnels through mesh processing and an improved point-to-plane matching technique, with an average error controlled within 0.38 m. The team also proposed a global positioning method based on solid-state LiDAR, which employs feature extraction [21], improved point cloud processing, and matching techniques, achieving high computational efficiency and low error rates. Improving accuracy and computational efficiency has always been a key focus of SLAM technology; however, the processing of large volumes of point cloud data may lead to performance bottlenecks, leaving room for further enhancement in computation speed [18,19]. The literature also indicates that in harsh underground environments, even well-protected vehicle electrical systems still exhibit high failure rates due to various causes [21], posing a threat to vehicle safety. To ensure a sufficient sensing range, multi-dimensional LiDAR is typically mounted on the vehicle roof. However, the addition of extra protective measures may compromise measurement accuracy, which poses a potential risk in roadways prone to roof leakage and rockfall.

In summary, existing technologies developed for underground environments have certain limitations. Considering the enclosed and low-dynamic characteristics of tunnel environments, this study draws inspiration from the automated guided vehicle (AGV) model and proposes a method for autonomous driving of trackless transport vehicles using a continuous, digitally controllable self-luminous object (hereinafter referred to as the light band) as the guiding element. The main contributions of this study are as follows:

1. Considering the characteristics of the underground mine environment, a light-band-guided autonomous driving method is proposed. A light band is installed at the top of the tunnel and controlled by a host computer, dynamically changing and emitting red, green, and blue colors in segments. The vehicle achieves autonomous driving by using two industrial cameras as the primary sensors to track the light band, along with a small number of single-point ranging radars for short-range safety detection. The self-luminous light band ensures stable capture by cameras even in low-light tunnel conditions. In addition to its flexibility, this approach is computationally efficient, cost-effective, and highly reliable.
2. A complete hardware and software system was developed for light-band guidance and vehicle navigation positioning. It consists of a server, a navigation control unit (NCU), and a GUI program. This system provides light-band guidance for vehicles and supports subsequent case studies.
3. An optimized image processing method is proposed for light-band recognition in mining environments. This method not only enables efficient and accurate color and trajectory recognition but also possesses certain anti-interference capabilities.
4. In a simulated tunnel environment, multiple scenario tests were conducted using a mining vehicle model to verify the effectiveness of the proposed method.

The structure of this paper is as follows: Section 2 reviews the relevant literature. Section 3 presents the method for implementing light-band guidance and navigation positioning. Section 4 presents the experimental section, including the detailed process of physical system construction and multiple scenario tests conducted to verify its effectiveness. Section 5 summarizes the conducted research.

## 2. Literature Review

### 2.1. Camera Image Acquisition in Low-Light Environments

Insufficient lighting in underground mine tunnels limits the use of cameras. Under low-light conditions, noise introduced by camera sensors increases significantly, leading to a decline in image quality [22]. These issues severely affect the accuracy of information acquisition through images. VSLAM (visual simultaneous localization and mapping) is also a mainstream SLAM technology that offers ease of installation and low cost compared with LiDAR [16]. However, it cannot handle low-light environments, and researchers typically do not consider using VSLAM in underground settings. Despite the various adverse effects of low-light conditions on camera performance, this challenge is inevitable across multiple domains. Therefore, low-light image enhancement (LLIE) plays a crucial role in computer vision and graphics. Extensive research has been conducted on image acquisition and object detection in low-light environments. Pixel-wise depth refinement [23] calculates the depth value of each pixel using deep learning models, providing detailed scene depth information to enhance the visibility and clarity of low-light images. LVIF-Net [24] aims to address the fusion of infrared and visible-light images under low-light conditions, significantly improving illumination and texture details in the fused images. The CLAHE [25] based low-light image enhancement method enhances contrast only in the luminance channel by separating the image’s luminance and chrominance channels, thereby avoiding color distortion. Jiang et al. [26] proposed a novel low-light image enhancement network, R2RNet, based on Retinex theory, which improves the image quality under low-light conditions by integrating spatial and frequency information.

The high-contrast characteristics of fluorescent labeling enable its widespread application in medical imaging and industrial inspection [27]. Self-luminous objects exhibit extremely high contrast against the background in low-light environments, allowing cameras to detect and recognize targets more easily, thereby improving detection accuracy.

### 2.2. AGV Navigation Technology

Automated guided vehicles (AGVs) are key technologies for achieving flexible production systems in Industry 4.0 [28,29,30]. AGVs use electromagnetic guidance, vision, and inertial navigation methods for path recognition and positioning. They are an essential part of modern logistics and manufacturing, performing tasks such as automated material handling through autonomous driving. AGVs were initially designed and developed for indoor applications in the early 1960s [11]. In the 1990s, AGV technology was applied in underground mining operations, where autonomous vehicles were guided by electromagnetic lines or reflective strips. Due to the need to reconfigure external facilities whenever transport routes change, the lack of flexibility led to research on global positioning for underground mining equipment gradually developed. However, in the harsh mining environment, the AGV model still has advantages due to its simple and reliable characteristics, with lower system complexity helping to reduce failure rates.

### 2.3. Path Planning Algorithms

Path planning [31,32] is a core issue in the fields of mobile robotics and autonomous driving. Path planning involves designing the optimal or feasible path for a mobile robot or vehicle from the starting point to the destination. This process requires consideration of the environmental map, obstacle locations, the motion capabilities of the target vehicle, and possible dynamic changes. Path planning first requires modeling the given environment and then, based on certain evaluation criteria (such as shortest distance, minimum time, etc.), finding a collision-free trajectory from the starting point to the target point.

Common algorithms used in path planning include the A* algorithm, Dijkstra’s algorithm, ant colony optimization, genetic algorithms, and particle swarm optimization (PSO) [31,33,34]. Each of these algorithms has its own advantages and is suited to different application scenarios. For example, in AGV systems, the vehicle’s operation route is often constrained by pre-set guiding devices, so the path planning problem can be simplified to selecting the optimal path based on known routes according to specific evaluation criteria. Dijkstra’s algorithm, as a typical shortest path search algorithm, is suitable for such scenarios. It connects various nodes and efficiently computes the shortest path from the starting point to all other nodes. In contrast, in SLAM-based application scenarios, path planning tasks typically need to adapt to dynamic environments, updating path planning in real time to effectively handle newly emerging obstacles and environmental changes [35]. Therefore, such path planning tasks require the algorithm to have strong real-time and adaptability capabilities, while also imposing higher computational demands on the hardware system.

## 3. Architecture of the Light-Band-Guided Autonomous Driving System

This chapter provides a detailed explanation of the system composition of the proposed method. The focus is on the tasks assigned to each component of the system, the image processing steps for light band recognition, and the principles and strategies for vehicle localization and traffic guidance.

### 3.1. System Components

**Figure 1:**
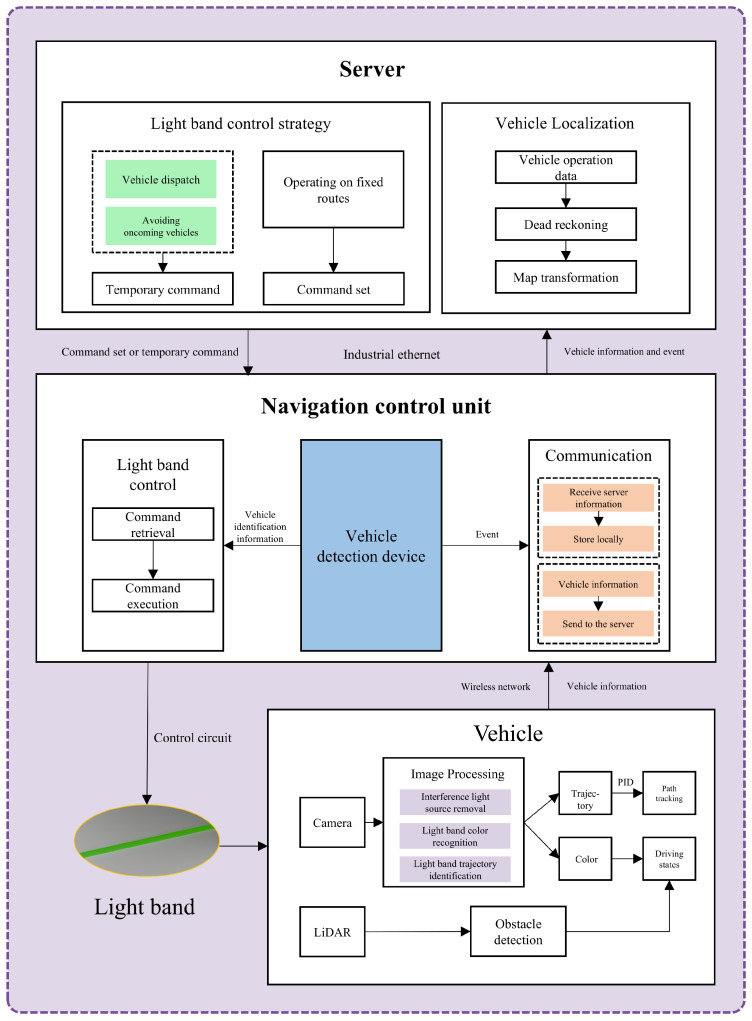
System architecture.

The system consisted of three main components: the server, the navigation control unit (NCU), and the vehicles. The server was located at the control center on the ground, responsible for generating the control plan for the light bands. The NCU was installed in the tunnel, receiving and executing commands issued by the server. The vehicles navigated along the light band and transmitted mileage data to the server. The server, situated on the ground, engaged in two-way communication with the NCU through an industrial Ethernet network. The NCU established a wireless network for the vehicles, allowing them to communicate with the server through this network. Figure 1 shows the system architecture.

#### 3.1.1. Functions of the Server Host

The server was the intelligent decision-making core of the system. The server undertook all key decision generation and computation tasks within the system, including path planning, the generation of light band control commands, and the coordination of communication with the NCU and the vehicles. It ensured that various components of the system operated intelligently according to the predetermined plan and dynamically handled emergencies or adjust commands, acting as the intelligent brain of the system.

The server could obtain the driving states of the vehicles, the image recognition of the light band, and other real-time data. These data included the vehicle’s speed, travel mileage, fuel status, etc., all of which were uploaded to the server via a wireless network. Based on these feedback data, the server could dynamically adjust the operating status of the system, such as optimizing path planning, reallocating light band commands, and even issuing temporary instructions to vehicles facing unexpected situations. This capability greatly enhanced the system’s flexibility and stability, ensuring that it operated efficiently and reliably in complex and dynamic mining environments.

The driving route of the vehicles was indirectly set by the light band, with the combination of light band trajectory and color determined by transportation task requirements. The light band control commands generated by the server were divided into command sets and temporary commands. For vehicles operating on fixed routes, the same light band control commands were repeatedly invoked over a long period, and these commands were grouped together as a command set. Once the command set was transmitted to the NCU, it was saved locally. Temporary commands were dedicated commands for managing unexpected situations, used to inform the NCU to initiate a command set or handle other urgent events. After being generated, temporary commands were sent instantly and waited for execution; the NCU removed them from local storage after executing once.

#### 3.1.2. Navigation Control Unit

The NCU is the core execution unit of the entire system, responsible for receiving and executing light band control commands generated by the server (command sets or temporary commands), interacting with vehicles, and controlling the light band. The NCU receives light band control commands from the server via industrial Ethernet, while converting the wired network into wireless signals transmitted into the tunnel for vehicles to send information to the server. The NCU was connected to the light band control circuit, capable of receiving vehicle identification information (a unique number assigned by the system to distinguish different vehicles), accurately identifying the vehicles and activating the correct light band to guide them.

The vehicle detection device was the component through which the NCU received and identified vehicle identification information. Its role was to capture vehicle identification information when vehicles passed by and transmit it to the NCU for handling. The performance requirements for the vehicle detection device included short-range communication and the necessity to be triggered when vehicles reached a specific location, typically installed at the connection point of two light bands or near intersections. The communication address between each vehicle detection device and the navigation control unit (NCU) was fixed, allowing the NCU to accurately determine which particular vehicle detection device conveyed the vehicle identification information.

The command set received from the server included light band control information for the vehicles and vehicle identification information, among others. When a vehicle passed the detection device, it identified the vehicle identification information and sent it to the NCU, which retrieved the command set to find the corresponding light band control command, illuminating the correct light band to guide the vehicle. Temporary commands received from the server were used to respond to unexpected situations, such as vehicle dispatch and avoiding oncoming vehicles. Temporary commands took precedence over preset command sets in execution.

#### 3.1.3. Vehicles Guided by Light Bands

The vehicle terminal was the final link in the system, primarily responsible for conducting driving operations based on light band signals. The vehicle acquired light band images via a camera and utilized an onboard computer to execute an image processing algorithm for light band recognition. Based on the information conveyed by the light band, the vehicle adjusted its driving state and followed the light band trajectory. When an obstacle appeared ahead or behind, the system simply executed a stop-and-wait operation. Simultaneously, the vehicle uploaded its operational information (such as speed, distance traveled, fuel status, etc.) to the ground server via the network, allowing staff to monitor the vehicle’s operating status in real time. The specific structure of the model vehicle designed in this study will be detailed in Section 4.

### 3.2. Light Band Recognition Algorithm

In this study, the red, green, and blue colors of the light band were utilized as command signals, corresponding respectively to different vehicle driving states: red indicated stop, green indicated forward movement, and blue indicated reverse. The key to effectively guiding vehicles with light bands lay in the vehicle’s ability to accurately identify and track the light bands.

**Figure 2:**
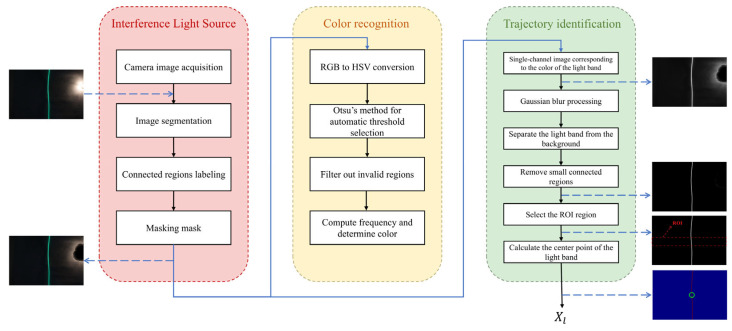
Light band recognition algorithm.

The image processing algorithm was built on the OpenCV library in Python (version 3.8.10). OpenCV is an open-source computer vision library that offers various image processing functions, such as image filtering, image transformation, image segmentation, and morphological operations [36]. To enable the autonomous tracking of the vehicle, the image processing process was mainly divided into three components: interference light source removal, light band color recognition, and light band trajectory identification, as shown in Figure 2. The following sections will elaborate on these three aspects.

#### 3.2.1. Interference Light Source Removal

Lighting equipment is typically installed in tunnels, with varying brightness at different locations. When the vehicle approaches a lighting fixture, the captured images may exhibit high-brightness areas, leading to inaccuracies in light band trajectory and color recognition [37]. Therefore, the illumination light source was regarded as an interference light. To eliminate interference from light sources, we converted the pixels generated by illumination into black to avoid their influence on subsequent recognition.

Image segmentation is the process of mathematically dividing an image into non-overlapping regions with similar properties, serving as an essential preprocessing step in computer vision [38]. Image segmentation was broadly classified into threshold-based methods, edge-based methods, region-based methods, clustering-based methods, and CNN-based methods [39]. Among these, threshold-based and edge-based methods were the most widely used, where one or more thresholds were set to divide the image into multiple regions, separating the target area from the background in the image. This image segmentation method was computationally simple and efficient. We eliminated interference light sources through threshold segmentation and masking. Before executing the color recognition algorithm, we first converted the color images obtained from the camera into grayscale images, then generated binary images through threshold segmentation, setting the aspect ratio and area thresholds to extract the connected regions that met the criteria. Using these connected regions, we created a mask that matched the size of the original image to obscure the high-brightness areas, thereby effectively excluding the influence of the interference light sources.

#### 3.2.2. Light Band Color Recognition

The color of the light band was identified based on the image hue characteristics. In the HSV image space, the three channels corresponded to hue, saturation, and value [40]. In HSV, brightness (V) was independent of hue (H) and saturation (S); lighting variations mainly affected the V channel, while the color information (H/S) remained relatively stable. Therefore, the HSV color space enabled more accurate identification of the light band color, minimizing interference from background highlights.

**Figure 3:**
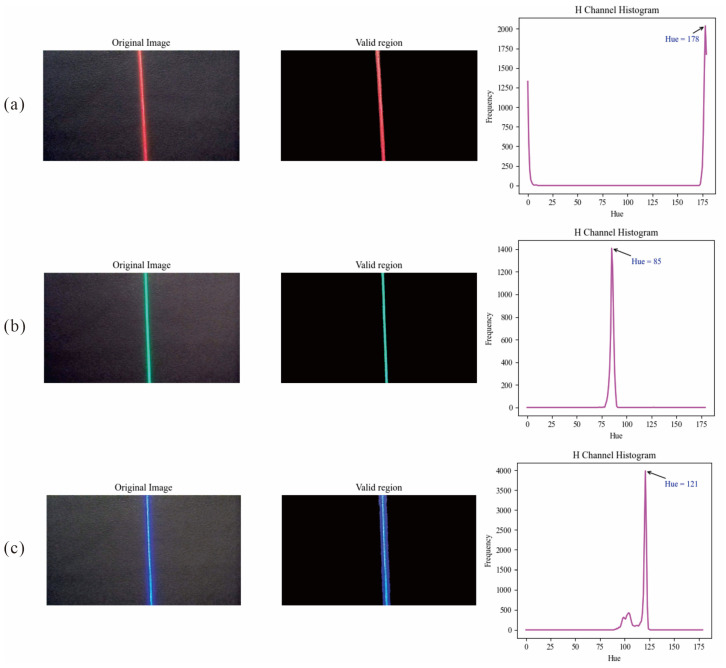
Valid region and H channel histogram. (**a**) Red light band; (**b**) Green light band; (**c**) Blue light band.

We identified the light band color by locating the hue with the highest pixel count. First, the image captured by the camera was in RGB format, which needed to be converted to HSV format. Next, the Otsu [41] algorithm was used to automatically determine the threshold for filtering low-brightness regions. Otsu is a method for determining the optimal threshold for image binarization. It operates by dividing the grayscale image into two classes based on pixel intensity levels and selecting the threshold that best separates them. Additionally, areas with weak color were filtered out by applying a saturation threshold of 50. The pixels covered by the mask used to remove interference light were excluded as well, and the remaining area was considered the valid region. Next, hue ranges corresponding to the three colors were selected. In OpenCV, the hue component in the HSV color space ranges from 0 to 179. Different colored light bands exhibited distinct hue values in the H channel. Considering the standard hue values of red, green, and blue, as well as the actual light band settings, the hue ranges were set as follows: red was [0, 10] ∪ [170, 179], green was [50, 90], and blue was [100, 140]. The valid regions (where color was preserved) and invalid regions (converted to black) for different colored light bands, along with their hue histograms, are illustrated in Figure 3. Finally, within the H channel, the frequency of each hue value was computed over the valid region; the hue interval containing the most frequent value was identified as the light band color.

(1) $$C=\left\{\begin{array}{c} r\;\;\left(0\leq H\leq 10\right)\\ r\;\;\left(170\leq H\leq 179\right)\\ g\;\;\left(50\leq H\leq 90\right)\\ b\;\;\left(100\leq H\leq 140\right) \end{array}\right\}$$

*C* is the final color recognition result, taking the value of *r*, *g*, or *b*, corresponding to red, green, and blue, respectively. H represents the hue value with the highest frequency.

#### 3.2.3. Light Band Trajectory Identification

Light band trajectory identification essentially involved separating the light band from the background formed by other objects for further processing. In this step, we processed the RGB image after applying the masking operation from the interference light source elimination algorithm. First, a single-channel image corresponding to the identified light band color was extracted. Subsequently, Gaussian filtering was performed on the single-channel images to improve their smoothness and noise immunity. Next, after filtering, threshold segmentation was applied to generate a binary image. Finally, small connected regions were removed. The use of a single-channel image significantly enhanced the noise resistance of threshold segmentation, which effectively separated the light band from the background and consequently improved recognition accuracy. We established a region of interest (ROI) [42] and searched for the center point of the light band within this region (this was the target point that the vehicle tracked). The specific operational steps were as follows: we polled each row in the ROI for white pixel points, grouping the horizontal coordinate values of these pixels by row and extracting the median of the horizontal coordinate values for each group of white pixel points, and then computed the average of all medians to obtain the horizontal coordinate of the light band’s center point.

### 3.3. Vehicle Positioning

#### 3.3.1. Overview of Dead Reckoning Technology

Dead reckoning (DR) is a navigation method used to determine the position of a moving object, widely applied in various environments where external positioning signals cannot be relied upon, such as underground mines, maritime, and aerial navigation. The basic principle of dead reckoning is to accumulate calculations over time based on known initial positions, the object’s speed [43], and heading information to determine the current position. Specifically, the displacement of the object can be calculated as the product of speed and time, while the direction is corrected by the heading angle. By using sensors such as accelerometers, gyroscopes, and magnetometers [44], real-time motion data of the object can be obtained and combined with dead reckoning models for comprehensive calculations. However, dead reckoning technology is characterized by cumulative errors, meaning that the positional estimation errors will continuously accumulate over time [45]. Therefore, dead reckoning is often used in conjunction with other positioning methods (such as GPS and visual positioning) to correct errors using external information, thus improving overall positioning accuracy and reliability.

#### 3.3.2. Longitudinal Positioning and Error Correction Methods for Vehicles

**Figure 4:**
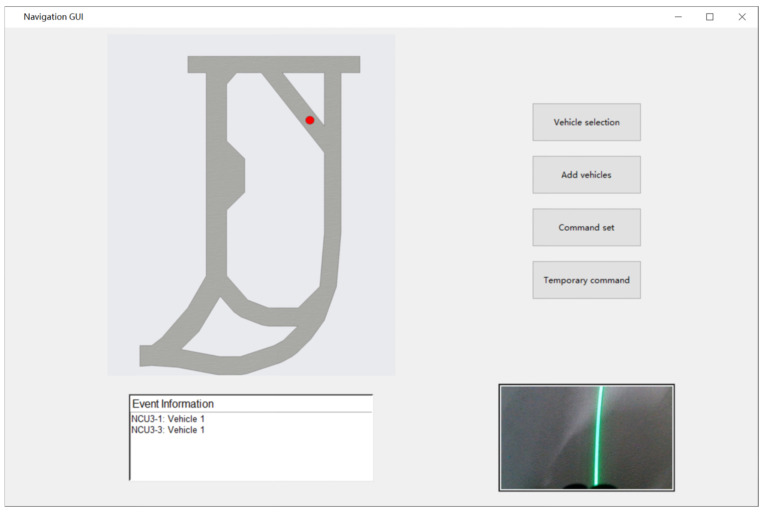
GUI interface.

Vehicle positioning was based on dead reckoning technology. Since the lateral error of the vehicle primarily depended on its tracking accuracy of the light bands, the localization calculation mainly focused on the longitudinal displacement of the vehicle. In this process, dead reckoning technology was employed, considering only the vehicle’s driving direction and distance within the tunnel. The position estimation of the vehicle used the driving distance. Using the initial position coordinates of the vehicle as a reference, the next position coordinates were calculated based on the driving distance and direction, which were then mapped to the floor plan of the tunnel. We developed a GUI program that ran on the server to display the real-time position of the vehicle. In the GUI interface, the vehicle’s current position was represented as a circular icon, as depicted in the actual positioning screen shown in the Figure 4.

Since the result of the current position estimation was based on the accumulation from the previous step, the error would gradually accumulate during prolonged travel, leading to divergence in positioning accuracy. To overcome this issue, we utilized fixed vehicle detection devices installed within the tunnel. When a vehicle triggered a detection device, the NCU received the vehicle identification information and uploaded it to the server, which immediately refreshed the vehicle’s coordinates at the triggered detection device, effectively eliminating cumulative errors.

### 3.4. Vehicle Traffic Guidance Management and Priority Policy

Due to ground pressure, rock properties, and cost considerations, the tunnel width generally allowed only one vehicle to pass. During encounters in passing lanes or avoidance chambers, yielding rules must be followed [46]. When vehicles met on a certain section, we allocated passing priority based on the principle that loading vehicles yielded to passenger vehicles, lighter vehicles yielded to heavier ones, uphill vehicles yielded to downhill ones, and vehicles outside the intersection yielded to those inside the intersection. When the server operator manually scheduled a vehicle to travel to a fixed location, the scheduled vehicle had the highest priority [47], and other vehicles along the way should nearby enter avoidance chambers or stop outside tunnel intersections to wait.

**Figure 5:**
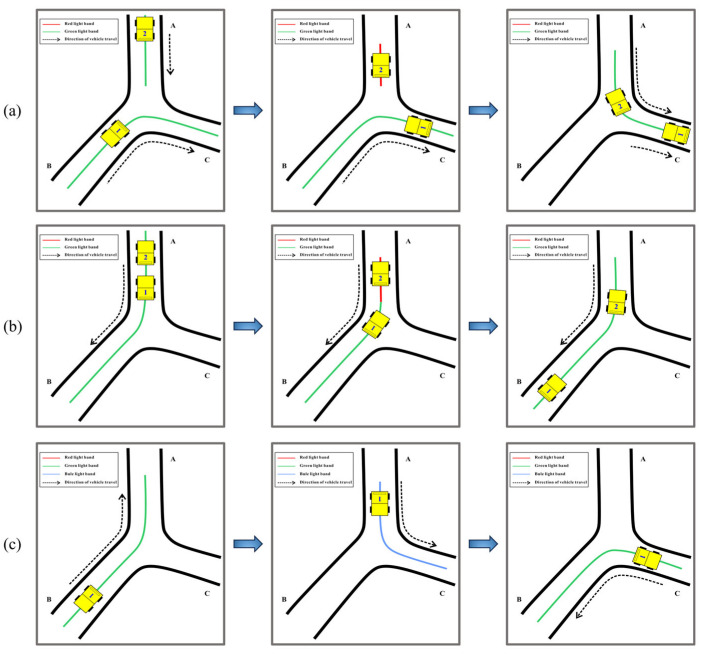
Light band working mode. (**a**) Encountering at an intersection; (**b**) Maintaining a safe distance; (**c**) Vehicle turning around.

The operational rules for underground vehicles were managed through light band control commands generated by the server, expressed in the form of light bands. Under the control of the NCU, the light bands could be illuminated or extinguished in segments and change colors as needed. During system operation, as a vehicle traveled a certain distance, the corresponding light band would light up to meet the different passage requirements of the vehicle. To illustrate the working mode and functionality of the light bands, we took an example of a tunnel intersection with three junctions. As shown in Figure 5, (a) Vehicle 1 entered the tunnel intersection first and had a higher passing priority. The red light band illuminated, causing vehicle 2 to stop at the junction and wait for vehicle 1 to pass. Only after maintaining a safe distance could vehicle 2 continue along its original route. (b) Vehicles 1 and 2 had the same driving route, but the distance between the two vehicles was too small. The red light band was activated for vehicle 2, causing it to stop in place while vehicle 1 continued its normal operation to maintain a safe distance. (c) To enable vehicle 1 to turn around at this location, the green light band directed the vehicle from junction B to junction A. Once there, the blue light band lit up, allowing the vehicle to reverse into junction C, and then the green light band illuminated again to complete the turnaround maneuver.

## 4. Experimental Design and Effectiveness Verification

Physical simulators provide substantial convenience in the fields of autonomous driving and robotics research [48,49]. Real-world robotic systems are often expensive, fragile, and limited in quantity. Simulators provide a low-cost environment that enables users to access various robotic platforms without risking damage to physical systems. However, most of these simulators are not designed for mining scenarios, with a primary focus on sensor applications or kinematic modeling. This study focused more on the logic behind light-band guidance, and simulators struggled to evaluate the effects of random environmental factors on the system. Testing with real vehicles in actual underground tunnels posed uncontrollable risks. Therefore, this study employed scaled-down physical models for real-world testing within a laboratory setting.

### 4.1. Construction of the Simulated Tunnel

Based on the conditions of a certain mine tunnel, a simulated area of 9 m × 4.5 m was constructed in the laboratory at a 1:12 scale, with multiple tunnel intersections, passing lanes, and avoidance chambers. The light bands were laid in the area to guide the vehicles, with the bands placed on the floor for observation purposes (they should be installed on the tunnel ceiling in the mine). During the installation, the safety distance between the vehicle and the tunnel walls during travel was considered, with the light band representing the vehicle’s centerline. The light bands were equipped with the necessary control circuits, including power supplies and relays. Simultaneously, multiple NCUs and vehicle detection systems needed to be installed in the area.

**Figure 6:**
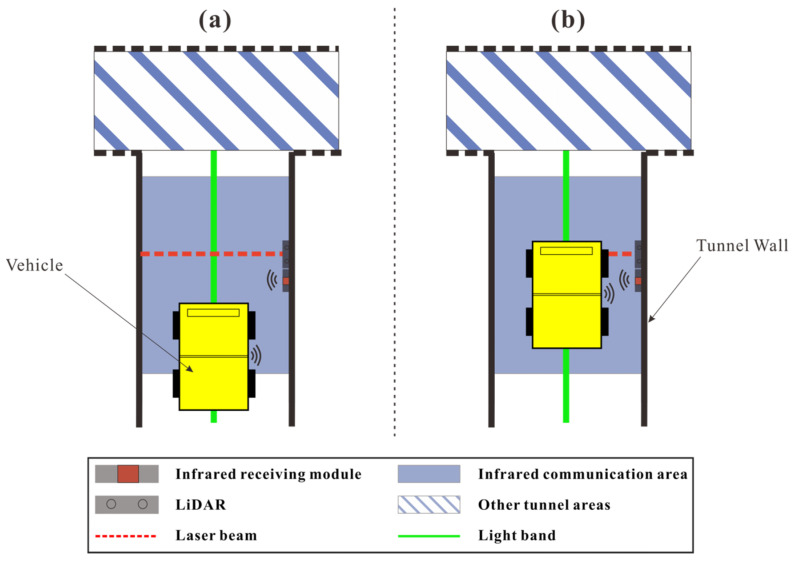
Schematic of the vehicle detection device. (**a**) Communication established but the detection device is not triggered; (**b**) The detection device is triggered.

We used a microcontroller to build the NCU. A microcontroller with rich interfaces was used as the main body, equipped with a network communication module to receive and send network information. It was also connected to the light band control circuit, where it controlled the on/off and color changes of the light band. Five NCUs were installed in the simulation area, with each typically responsible for managing one tunnel intersection. The management range depended on the number of interfaces and the wiring length. In special sections, the NCU’s management range was broader. We used a vehicle detection device composed of a point laser rangefinder and an infrared receiving module. The infrared receiving module was placed alongside the point laser rangefinder, and the infrared transmitting and receiving modules communicated within a certain range. The detection device was triggered when the infrared signal was received and the laser rangefinder’s beam was blocked. As shown in Figure 6a, the blue area represents the communication zone of the infrared module. The vehicle’s current position established infrared communication with the vehicle detection device, but the laser beam was not blocked, and the detection device was not triggered. As the vehicle continued to move forward and reached the position shown in Figure 6b, the position of the laser beam became the “detection line”. When the vehicle crossed this line, the detection device was triggered.

**Figure 7:**
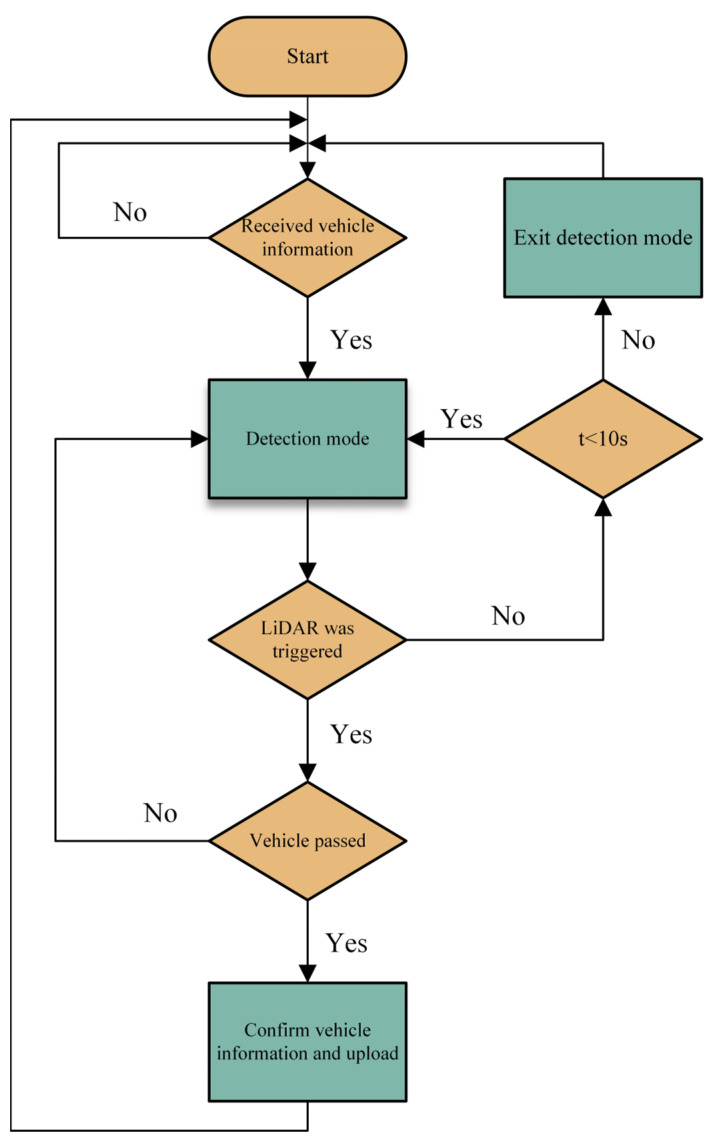
Operation flowchart of the vehicle detection device.

The operational logic of the vehicle detection device is shown in Figure 7. After receiving the vehicle’s infrared information, the device entered detection mode, and the laser rangefinder began to operate. If no object was detected within 10 s, the system exited detection mode; if an object was detected, further type analysis was conducted. If the distance data suddenly fluctuated and quickly ended, it may have been a pedestrian passing by; if the data stayed within a certain range for a period of time (due to the vehicle’s length causing the reading to change over time), it was judged as the vehicle reaching the detection line. The NCU then identified the vehicle’s identification information and uploaded the event information of the vehicle triggering the detection device.

The layout of the light bands and vehicle detection devices could be flexibly adjusted based on the vehicle’s running route. In the simulated tunnel area, we set up vehicle detection devices at every intersection based on the various driving routes and operational states of the vehicles to accurately capture the vehicle’s position and identity information. However, in actual underground mining operations, due to the relatively simple and single nature of the vehicle routes, it was unnecessary to install vehicle detection devices at every intersection. To improve efficiency, detection devices were only required at key nodes along the driving route, where the light bands needed to change position. This optimized layout not only met the vehicle guidance requirements but also effectively reduced equipment installation and maintenance costs.

### 4.2. Fabrication of Vehicle Models

#### 4.2.1. Vehicle Hardware Components

**Figure 8:**
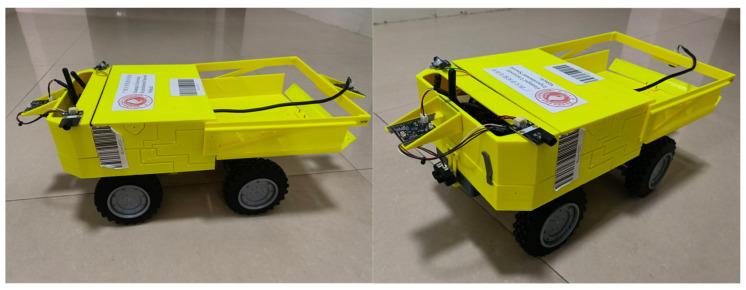
Appearance of the vehicle.

The 3D modeling design was conducted at a 1:12 scale, and a mining truck model is produced using a 3D printer. The power system included an reduction motor with an odometer, a servo motor, and a 12V lithium battery. The geared motor outputted power to the front and rear axles through a transfer case, enabling four-wheel drive, with a differential device in the axles. Two servo motors were located above the front and rear axles and controlled tire deflection through tie rods to achieve steering. The hopper could tilt backward to unload materials, and its lifting and lowering were controlled by a servo motor installed beneath the hopper, driving a rocker arm. The appearance of the vehicle model is shown in Figure 8.

The vehicle model was equipped with an NVIDIA Jetson Orin Nano (4GB) (hereinafter referred to as the onboard computer) to run image processing and navigation programs. Two cameras were installed at the front and rear of the vehicle, with their centers aligned with the vehicle’s central axis and a resolution of 2 megapixels (1920 × 1080). Two point laser rangefinders were used to detect obstacles in front of and behind the vehicle. Infrared signal transmission modules were installed on both sides of the vehicle, for the system to receive vehicle identification information.

#### 4.2.2. Automatic Control Mechanism for Vehicle Navigation Guided by Light Bands

The reduction motor was responsible for controlling the vehicle driving states, while the servo motor was responsible for steering the vehicle. The light band color recognition result C was mapped to different control instructions, which then controlled the operation of the reduction motor and adjusted the vehicle operational states. The light band trajectory recognition result provided the horizontal coordinate value $X_{l}$ of the light band’s center point in the image, which was used for vehicle tracking of the light band trajectory. During operation, the projection of the camera’s center point on the ground (the tunnel ceiling in the mining environment) should coincide with the light band trajectory to ensure the vehicle followed the light band trajectory. When the vehicle deviated from the light band trajectory, the system adjusted using the PID control algorithm. The PID control algorithm, due to its ease of implementation and mature technology [50,51], has become the most widely used automatic feedback system and is suitable for real-time tracking of the light band trajectory. The mathematical expression of PID control is

(2) $$u_{t}=K_{p}e\left(t\right)+K_{I}\int_{0}^{t}e\left(\tau \right)d\tau +K_{D}\frac{d}{dt}e(t)$$

Here, $u_{t}$ is the output; $K_{p}$, $K_{I}$, and $K_{D}$ are the proportional, integral, and derivative gains, respectively; and e(t) is the system error.

**Figure 9:**
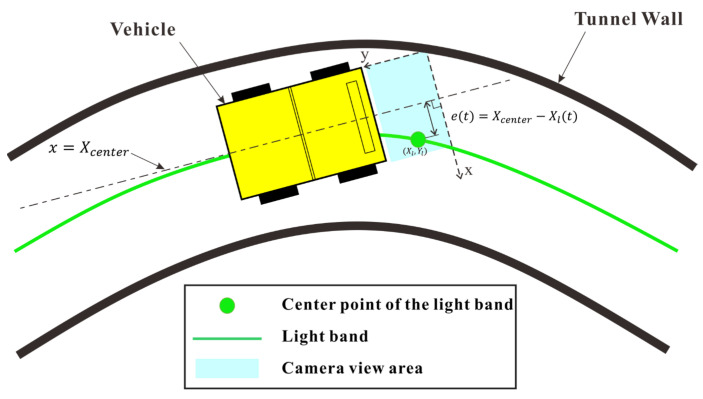
Principle of vehicle tracking the light band.

The principle of the vehicle’s automatic tracking of the light band is shown in Figure 9, where the blue area represents the camera’s field of view. The center point of the camera’s image ($X_{center}$, $Y_{center}$) coincided with the vehicle’s centerline, which can be represented as $x=X_{center}$. The error $e\left(t\right)$ in vehicle tracking of the light band can be approximated as

(3) $$e\left(t\right)=X_{center}-X_{l}$$

This error was used as the input to the PID algorithm, and the output was mapped to the pulse count to control the rotation of the servo motor, making $e\left(t\right)$→0, thereby maneuvering the vehicle to follow the light band.

The camera input was dynamically switched based on the vehicle operational states. When the vehicle was moving forward or stopped, the system used the image captured by the front camera as the input for image processing. When the vehicle was reversing, the image from the rear camera was used as the input. This switching mechanism ensured that during forward movement, the center point of the front camera remained aligned with the light band, while during reversing, the center point of the rear camera was aligned with the light band.

#### 4.2.3. Vehicle-Side Multithreading Processing and Emergency Response Design

To ensure precise driving of the vehicle under light band guidance, while also being capable of handling emergencies, the system employed multithreading processing technology on the vehicle side to achieve real-time data processing and emergency response [52,53]. The three parts of image processing were encapsulated in independent subprograms, with parallel computation achieved through shared image variables. The multithreading design not only improved the computational efficiency of the system but also ensured the real-time performance of each processing module. The main program was responsible for controlling the vehicle actuators and network communication, uploading the vehicle’s driving distance and status data in real time.

In the simulated environment, the vehicle mainly faced two typical situations that required autonomous responses: (1) Encountering an obstacle: When the laser rangefinder detected an obstacle within 20 cm in front of the vehicle, the system triggered a deceleration mechanism. If the obstacle distance was further reduced to 10 cm or less, the vehicle would perform an emergency stop, wait in place, and upload the event information to the server. When the obstacle was removed or the distance returned to a safe range, the vehicle would restart after 5 s and resume normal driving. (2) Loss of light band: In the absence of obstacles, if the image processing did not detect the light band in a certain frame, the vehicle would not stop immediately but would first decelerate and continue processing the next five frames of images. If the light band was still not detected, the system would trigger an emergency stop and upload the event to the server. Once the light band reappeared in the camera’s field of view, the vehicle would automatically restart. All similar event processing information would eventually be transmitted to the server for real-time monitoring and subsequent handling by the operator.

### 4.3. Image Processing Performance Test

**Figure 10:**
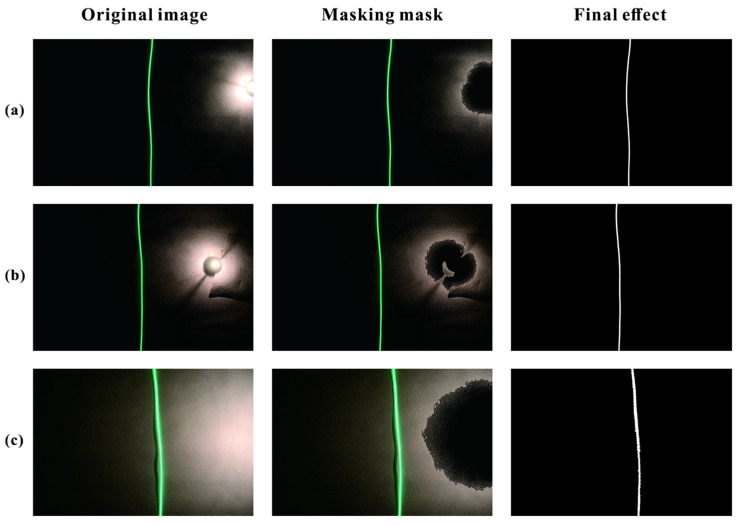
Effect of excluding interference light sources. (**a**) The small incandescent lamp is partially within the camera frame; (**b**) The small incandescent lamp is fully within the camera frame; (**c**) Light from the lamp enters the frame from the side.

In the simulated environment, we used a small incandescent lamp as a lighting source to test the effectiveness of excluding interference from light sources. A small incandescent lamp with a rated voltage of 6 V and a rated power of 5 W was selected as the simulated light source. It was fixed on the ground and the wall of the simulated tunnel to create several states, including direct, side, and diffuse reflections from the small lamp on the vehicle camera. The vehicle’s camera captured images, and the above algorithms were run. Subsequent light band color recognition and trajectory recognition operations were performed on the masked images. The experimental results are shown in Figure 10. The bright area formed by the lamp’s illumination did not affect the recognition of the light band trajectory.

### 4.4. Tracking Accuracy and Repetitive Localization Accuracy

**Figure 11:**
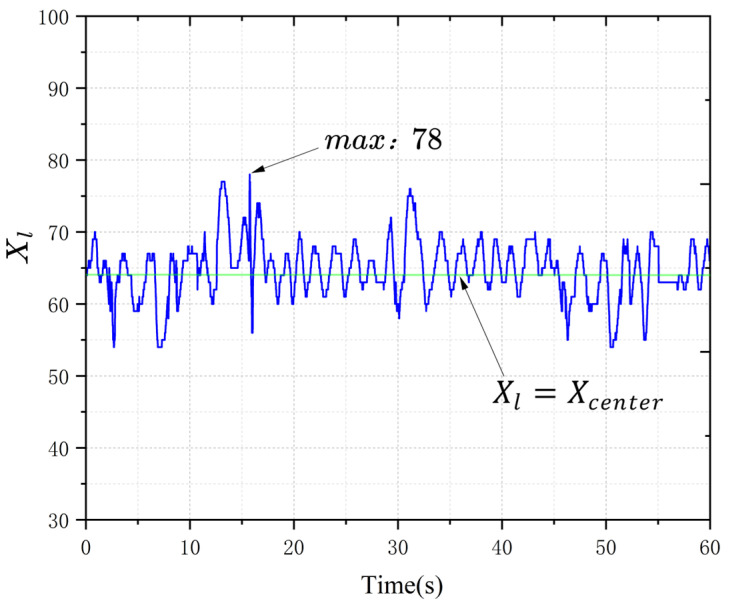
Vehicle tracking light band error.

In the simulated environment, a manually generated command set was used to guide the vehicle along a predetermined route. The horizontal coordinate $X_{l}$ of the center of the light band trajectory, identified by the light band recognition algorithm during the vehicle’s movement, was extracted, as shown in Figure 11, which is a line plot of $X_{l}$ over time t. In the light band recognition phase, the camera image was compressed to a width of $W_{pixels}=128$ pixels, with the X-coordinate of the image center point $X_{center}=64$. Theoretically, the camera remained vertically aligned with the ground at a fixed distance; measurement indicated the actual width of the vehicle’s camera view was 175 mm, so the actual distance per pixel was $D_{pixels}$ = 1.36 mm. In the figure, the maximum $X_{l}$ value was 78, where the absolute value of the deviation $e=X_{center}-X_{l}$ reached its peak, resulting in a maximum lateral deviation of 19.04 mm on one side of the vehicle. Due to the strength of 3D-printed materials and assembly precision, there was no straightforward linear relationship between the tire deflection angle and actual steering capability of the vehicle model, resulting in a small, continuous oscillation in the tracking error. Motion control of the vehicle was not the primary focus of this study; we only required the vehicle to have basic light band tracking capability. In practice, the vehicle maintained a sufficient safety distance from the tunnel walls, with no collisions occurring.

**Figure 12:**
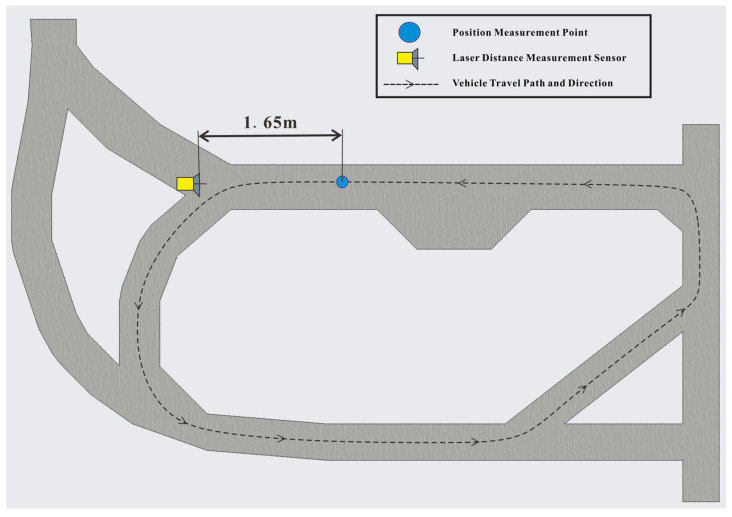
Measurement of vehicle repetitive positioning accuracy.

Using a manually generated set of commands, the vehicle was guided along a circular route within the site, with a total length of 17,505 mm, and a high-precision laser rangefinder sensor was installed at a fixed position, as shown in Figure 12. By running additional programs, the NCU drove the laser rangefinder. Whenever the vehicle reached a position measurement point in the navigation system, the server’s GUI program issued a command, and the NCU collected measurement data from the laser rangefinder while recording the time, to test the repetitive positioning accuracy of the navigation system. The actual distance from the position measurement point to the laser rangefinder was 1.65 m. The vehicle ran for 30 laps, and the measured data are shown in Table 1.

The measurement data showed that the repeated positioning distances were relatively concentrated, indicating high precision. Analysis shows that the larger individual errors in the table were caused by the vehicle’s lateral displacement, which caused the laser beam to hit protruding structures on the vehicle’s body. Due to the NCU’s positioning refresh function, the vehicle’s positioning error would only accumulate between the two vehicle detection devices of the NCU and would not accumulate over time in the global positioning system.

### 4.5. Case Study

Artificially generated light band control commands were transmitted from the server host to the NCU to execute the following scene operations. Scene 1 was a segment of the vehicle’s fixed route operation mode, while scenes 2 and 3 represented temporary events. Scene 1 was a segment of the vehicle’s fixed route operation mode, while scenes 2 and 3 represented temporary events.

**Figure 13:**
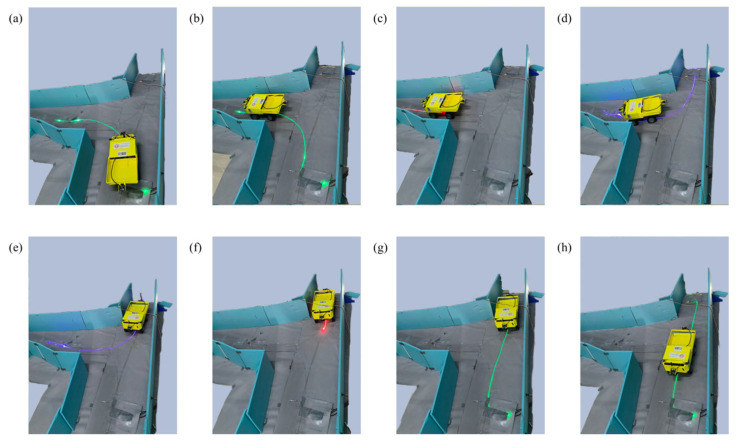
Vehicle U-turn at intersection. (**a**) Vehicle at initial position; green light band to left intersection is activated; (**b**) Vehicle is detected by the sensor; (**c**) Red light band is activated; vehicle stops; (**d**) Red light band is turned off and blue light band is activated for reversing; (**e**) Vehicle reverses and is detected by the sensor; (**f**) Red light band is activated; vehicle stops; (**g**) Green light band is activated for forward movement; (**h**) Vehicle moves forward and completes the U-turn.

Scene 1: The vehicle made a U-turn at an intersection while traveling along a fixed route, as shown in Figure 13. (a) The vehicle was at the position shown, and the NCU turned on the green light band leading to the left intersection according to the command set. (b) The vehicle followed the light band and moved forward to the position shown, where the vehicle detection device identified the vehicle. (c) The NCU turned on the red light band for the vehicle, and the vehicle stopped. (d) After 1.5 s, the NCU turned off the red light band and activated the blue light band, and the vehicle followed the light band in reverse. (e) The vehicle reversed to the position shown, where the detection device identified the vehicle. (f) The NCU turned on the red light band, and the vehicle stopped. (g) After 1.5 s, the NCU turned on the green light band, and the vehicle followed the light band to move forward. (h) The vehicle passed through the tunnel intersection and completed the U-turn.

**Figure 14:**
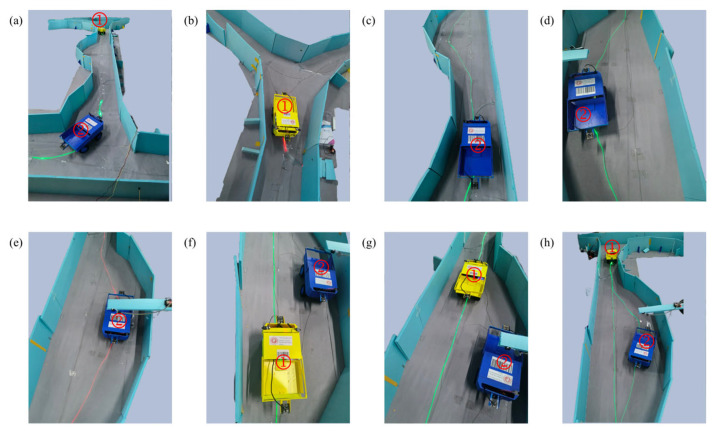
Two vehicles meeting. (The labels “1” and “2” represent Vehicle 1 and Vehicle 2, respectively) (**a**) Two vehicles approach; Vehicle 2 is directed to passing lane, Vehicle 1 is stopped; (**b**) Vehicle 1 stops before the passing lane; (**c**) Vehicle 2 is guided into the passing lane; (**d**) Vehicle 2 arrives and is detected; (**e**) Vehicle 2 stops and waits in the passing lane; (**f**) Vehicle 1 resumes its route; (**g**) Vehicle 1 passes through the passing lane; (**h**) Vehicle 2 exits the passing lane and resumes its route.

In scene 2, two vehicles running along their respective fixed routes met at a single-lane, bidirectional road section. The yellow vehicle was vehicle 1, and the blue vehicle was vehicle 2. The scene was set such that vehicle 1 had higher priority than vehicle 2, and vehicle 2 must proceed to the passing lane to avoid the encounter. As shown in Figure 14, (a) the two vehicles were approaching each other in the same section of the tunnel. The server determined that the vehicles would soon meet based on their positions and directions, assessed priority, and required vehicle 2 to enter the passing lane to avoid the encounter. Simultaneously, a temporary command was sent to the NCU to stop using the relevant commands in the vehicle’s fixed route command set for vehicle 1 and to turn on the red light band when it reached the next vehicle detection device. (b) Vehicle 1 triggered the vehicle detection device, and the NCU turned on the red light band to stop vehicle 1 before the passing lane, uploading its arrival information to the server. (c) After receiving the parking information from vehicle 1, the server sent the second temporary command, stopping the relevant control commands for vehicle 2 in the fixed route command set and activating the green light band leading to the passing lane, guiding vehicle 2 to the passing lane. (d) Vehicle 2 reached the passing lane, and the vehicle detection device in the passing lane recognized vehicle 2, uploading its arrival information to the server. (e) The server sent the third temporary command to the NCU, turning on the red light band. Vehicle 2 stopped and waited in the passing lane, and then the light band was turned off (due to limitations in the light band layout, the closed light band was used to replace the red light band for stopping functionality, based on the vehicle’s safety design). (f) The server sent the fourth temporary command to the NCU, restoring the relevant control commands for vehicle 1 in the command set. Vehicle 1 resumed its fixed route operation. According to the commands in the command set, the NCU turned on the green light band for vehicle 1, and the vehicle moved forward. (g) Vehicle 1 passed through the passing lane. (h) Vehicle 1 triggered the vehicle detection device at its current location. After receiving the information, the server sent the final temporary command to the NCU, turning on the green light band to guide vehicle 2 out of the passing lane and restoring the relevant control commands for vehicle 2 in the fixed route command set. Vehicle 2 returned to its previous fixed route operation, completing the vehicle meeting process.

**Figure 15:**
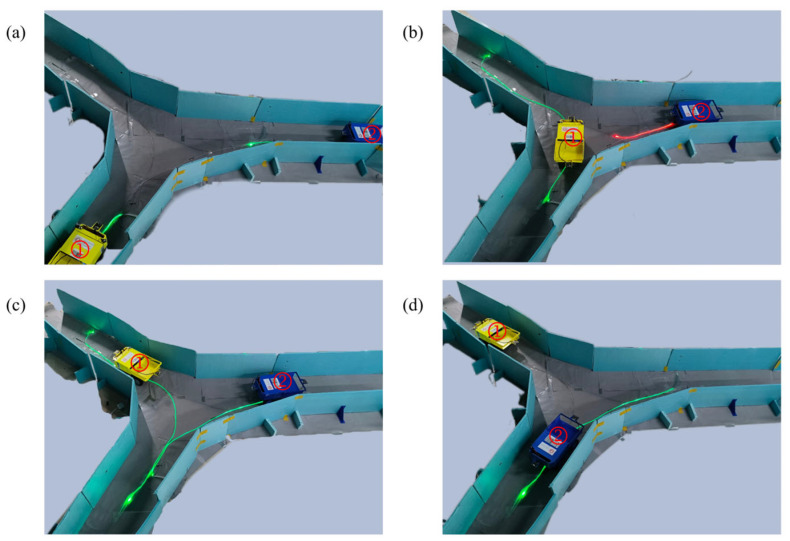
Vehicle avoidance at intersection for scheduled vehicles. (The labels “1” and “2” represent Vehicle 1 and Vehicle 2, respectively) (**a**) Vehicle 2 approaches the scheduled area; (**b**) Vehicle 2 is stopped by the red light band; (**c**) Vehicle 1 enters; Vehicle 2 resumes after command update; (**d**) Vehicle 1 stops at the target location to complete the task.

In scene 3, vehicle 1 received a scheduling command to go to the target location. The route from the starting point to the target location was called the scheduling route, and the vehicle receiving the scheduling command had the highest priority for passage. After the scheduling instruction was issued, the server first sent a temporary command to the NCU, terminating the relevant command set for the scheduling route, transmitting the new scheduling task command set, and running it. When running the scheduling task command set, the NCU first controlled the light band to guide other vehicles on the scheduling route to leave, then guided the scheduled vehicle to the target location while preventing other vehicles from entering. During the process of vehicle 1 heading to the target location, it met vehicle 2, which was on a fixed route. The system controlled vehicle 2 to wait outside the scheduling route. As shown in Figure 15, (a) at this point, vehicle 2 was about to enter the scheduling route area. The server had already terminated the use of the non-scheduled vehicle commands on the scheduling route in the fixed route command set through a temporary command and had turned on the red light band for all other vehicles. (b) Vehicle 2 triggered the vehicle detection device, and the NCU turned on the red light band to prevent it from entering the scheduling route area. (c) Vehicle 1 triggered the vehicle detection device at its current location, where no other vehicles were operating in the closed section. The NCU activated the green light band according to the scheduling task command set and uploaded vehicle 1′s information; after the server received the information, it sent a temporary command to terminate the NCU’s scheduling task command set, restoring the fixed route command set for the scheduling route area, and vehicle 2 resumed its operation on the fixed route. At this point, vehicle 1 had not yet reached the target destination of the current scheduling task. (d) The server used vehicle 1′s odometer information for dead reckoning to locate vehicle 1. When vehicle 1 reached the target location, the server sent the final temporary command to the NCU and turned off the light band, and vehicle 1 stopped moving forward, completing the scheduling task.

## 5. Discussion

This paper proposes an autonomous driving system guided by light bands, which was validated through model-based experiments. A complete hardware–software system was developed to support dynamic light band control, enabling color switching among three states and segment-based activation. Vehicles equipped with cameras captured images containing the light band, and image processing algorithms were applied to identify both the color and the trajectory of the band. A PID controller was then used to steer the vehicle along the light band path, achieving autonomous driving within the tunnel environment. The system employed dead reckoning for vehicle localization, and the cumulative error was mitigated using an NCU-based vehicle detection device, ensuring high positioning accuracy. In laboratory tests, flexible variations in the light band effectively guided the vehicle to perform a variety of maneuvers. The light band recognition algorithm demonstrated high efficiency and accuracy, and the vehicle successfully completed complex tasks such as vehicle avoidance and traffic scheduling.

The light-band-guided autonomous driving system offers several advantages. Compared with traditional beacon-based methods, the light band system enables dynamic route adjustments without the need for manual repositioning of beacons. This improves vehicle flexibility and facilitates efficient handling of vehicle encounters and scheduling tasks. Since the light band is installed on the tunnel ceiling, it is less susceptible to contamination than barcode tags typically affixed to tunnel walls. Owing to its self-illuminating nature, the light band can be effectively captured by cameras even under low-light conditions. In contrast to SLAM systems that rely on LiDAR, cameras offer a more cost-effective solution. Additionally, the system delegates path planning and decision-making processes to a ground server, while the vehicle merely tracks the light band for navigation. This architecture reduces the computational burden on the vehicle side. In conventional SLAM systems, path planning is executed on board in real time, often accompanied by intensive point cloud processing, which imposes high performance requirements on the vehicle’s computing platform.

Currently, our tests have been limited to laboratory conditions, and the performance of the light band guidance system in real tunnel environments remains unverified. Suspended dust may introduce two primary challenges. First, dust may accumulate on the surface of the light band, reducing its brightness. This issue is relatively easy to address by equipping the vehicle with a brush mechanism mounted on top to perform real-time cleaning and maintain visibility. Second, in high-humidity environments, water vapor may condense into droplets on the camera lens, resulting in image blurring. Over time, these droplets may attract dust particles, forming mud stains. Therefore, in practical mine deployments, the camera must be equipped with a well-sealed protective housing, along with an automatic cleaning and wiping system installed externally, to ensure continuous image clarity. In future research, we will address these challenges by developing image defogging algorithms and automated cleaning devices. In terms of scheduling, AGV path planning algorithms can be referenced to develop server-based optimization scheduling strategies, further optimizing multi-vehicle collaborative scheduling and speed control functions, thereby enhancing the overall performance and applicability of the system in real-world applications. Furthermore, optimizing the existing PID control algorithm or developing new control strategies [54] can improve trajectory tracking performance and response speed.

## 6. Conclusions

This paper investigates the autonomous driving technology for trackless transport vehicles in underground mines. To achieve a balance between safety and cost-effectiveness, we propose an autonomous driving method based on light band guidance. The experiments showed that the optimized light band recognition algorithm not only possessed efficient color and trajectory recognition capabilities but also exhibited a certain degree of anti-interference performance. Additionally, the vehicle model was equipped with dual front and rear cameras, and the switching mode between the cameras was reasonable, effectively responding to changes in driving conditions. The light band control commands sent from the server side could be executed properly on the NCU, effectively controlling the light band. The results showed that the system, without relying on GPS, could effectively locate the vehicle using dead reckoning combined with light band guidance and eliminate accumulated errors through vehicle detection devices. Specifically, the vehicle could stably follow the light band guidance, handling various complex scenarios. This method can achieve stable and efficient autonomous driving for mining vehicles, providing new insights for autonomous driving research of trackless transport vehicles.

## Figures and Tables

**Table 1:** Measurement results.

Serial Number	Distance (mm)	Time (s)	Absolute Error (mm)	Relative Error (%)	Serial Number	Distance (mm)	Time (s)	Absolute Error (mm)	Relative Error (%)
1	1623	119.893	27	0.1542	16	1735	115.015	85	0.4856
2	1693	115.447	43	0.2456	17	1692	116.431	42	0.2399
3	1699	116.835	49	0.2799	18	1468	113.161	182	1.0397
4	1677	121.077	27	0.1542	19	1680	115.602	30	0.1714
5	1708	115.642	58	0.3313	20	1651	115.838	1	0.0057
6	1693	116.834	43	0.2456	21	1723	117.312	73	0.4170
7	1655	121.013	5	0.0286	22	1687	117.295	37	0.2114
8	1717	114.857	67	0.3827	23	1665	117.139	15	0.0857
9	1726	115.618	76	0.4342	24	1613	122.563	37	0.2114
10	1670	117.727	20	0.1143	25	1684	115.215	34	0.1942
11	1601	115.743	49	0.2799	26	1670	117.679	20	0.1143
12	1640	118.904	10	0.0571	27	1690	120.675	40	0.2285
13	1656	118.878	6	0.0343	28	1699	116.253	49	0.2799
14	1645	117.247	5	0.0286	29	1735	118.076	85	0.4856
15	1676	115.895	26	0.1485	30	1706	116.233	56	0.3199

## Data Availability

The original contributions presented in this study are included in the article. Further inquiries can be directed to the corresponding authors.
